# Case of an Affection of the Nervous System, Which Exhibited Some Unusual Phenomena

**Published:** 1826-09

**Authors:** 

**Affiliations:** the Middlesex Hospital


### NERVOUS AFFECTION.

*Case of an Affection of the Nervous System, which exhibited some unusual Phenomena.* Treated at the Middlesex Hospital, by Dr. Hawkins.

Elizabeth Small, aged twenty-one, was admitted into the hospital on the 28th of February, 1826, with severe headache accompanied by giddiness, under which she had laboured for three weeks, and for which, previous to her admission, she had been bled twice from the arm. The relief from these bleedings was inconsiderable, and of very short duration.

Some days after her admission, symptoms of paralysis began to show themselves, which were progressive in spite of the remedies employed. The bowels were well opened; leeches were applied to the temples; cupping and blistering to the back of the neck; and friction with liniments was employed to the paralysed parts. Various stimulant antispasmodics were subsequently administered, but with little or no effect in palliating the symptoms. It was now determined, as a *dernier ressort*, to affect the system with mercury; but this was accomplished with difficulty: the gums were sore for two days only, and no benefit resulted from the remedy. On the 22d of April, a seton was inserted into the nape of the neck; and she was discharged on the 2d of May, much relieved.

We relate this case on account of the paralytic symptoms, which were exceedingly curious and interesting, as well in a physiological as in a pathological point of view. The left arm and leg were partially paralysed, (indeed, the power of motion did not appear to be quite deficient in any part,) and had lost much of their natural sensibility. The muscles did not combine in their actions, and the will had but an imperfect influence over them; so that the power of directing the foot or hand was much impaired; she walked with difficulty, and dragged the affected leg after her.

But the most remarkable circumstance was, that, whilst the side of the face corresponding with the paralysed side of the body retained all its powers and endowments in a perfect degree, those of the opposite side were lost. The sensibility of the right side of the face was deficient, and she had no power of retracting this cheek. This was well seen when she made an attempt to blow her nose or whistle: the cheek then became convex, and the air escaped by the side of the mouth, in consequence of the want of muscular resistance. There was very little motion in the nostril of this side, and the little which was observed appeared to be derived from its connexion with the other ala: it could not be drawn down and narrowed, as in the act of smelling. She could not close the right eyelid: it fell over a part of the cornea, which it appeared to do by its own weight; thus exhibiting the appearance technically termed *ptosis*. The winking motions were lost: on threatening the eye, no motion of the lid was observed, excepting a slight tremor, in sympathy with the other eyelid.

The senses corresponding with the paralysed side of the face were all affected. The acoustic nerve did not perform its office, and the sense of hearing on this side was very imperfect. The state of the olfactory nerve was not so easily examined; for, as smelling is a compound operation, so the paralysis of the portio dura interfered with our observations. But the most curious phenomenon was that exhibited by the gustatory nerve: the sensibility of the tongue on this side was not destroyed, but the finer sense of taste was quite deficient; thus countenancing the opinion that, if taste is merely a modification of touch, it is, perhaps, of a more delicate and refined nature. The power of motion in the tongue communicated by the ninth nerve was impaired. We were informed that the right side of this organ had been formerly paralytic: this, however, was not the case when in the Middlesex Hospital; she could move it to either side, and this might appear at first view to be conclusive. But the finely regulated motions of the tongue, necessary for perfect and distinct articulation, were deficient: she articulated imperfectly, and babbled like a drunken person; or, to use a common expression, her tongue appeared to be too large for her mouth.

The state of the eye presented many curious appearances. The retina manifested very little sensibility to light: thus, the stimulus to the action of the recti muscles was removed, and the eyeball was no longer under their control, but was delivered up to the obliqui, and a confirmed squint resulted. She stated that, when her sight in this eye first began to be impaired, she had double vision; but this gradually subsiding, was followed by a loss of sight in this eye.

In order to ascertain whether the motor oculi was implicated, it was necessary to close the sound eye, and thus to call the attention of the patient to the direction of the eye affected; by this method the recti were brought into action. It was now evident that the recti muscles, with the exception of the abducens, had much of their power remaining: the latter muscle, however, appeared to be completely paralysed. The inferior oblique muscle seemed also to be only slightly affected; for the eyeball was turned up in unison with that of the opposite side, although less perfectly.* When the left eye was open, the right and weak eye was not used. The vision was single, as in common cases of squinting.

It seemed not improbable that double vision would again be produced when the healthy action of this eye should be about to be restored, should this fortunate result ever be accomplished. This we afterwards found was actually the case.

Another circumstance worthy of remark was, that this patient was unconscious of her inability to move the right side of her face, until it was pointed out to her; and this seems invariably to be the case in all those instances where the portio dura and the fifth nerve, or the fifth nerve alone, are affected: that is to say, in all recent cases. Those labouring under this affection, when informed of the loss of power in their faces, are much chagrined, especially if it be put to the proof by looking in the glass. The above circumstance forms a good illustration of Mr. Bell's views of the Nervous Circle, lately published in the Philosophical Transactions; and, indeed, the whole case becomes very interesting when considered with reference to his doctrines.

To account for the paralysis of the face and body being on different sides, is perhaps impossible. Lieutaud, Winslow, and other anatomists, have indeed described the decussation of the pyramidal bodies, and Vicq d'Azyr that of the spinal marrow; all of which every accurate anatomist acknowledges. But the decussation of the brain remains still to be proved; and it is only by accurate observation that we are likely to gain any insight into this obscure subject.

* See Mr. Bell's remarks on the use of the Obliqui, in his Exposition of the Nervous System.

